# A self-directed digital exercise program for hip osteoarthritis (“My Hip Exercise”): protocol for a randomised controlled trial

**DOI:** 10.1186/s12891-023-07009-1

**Published:** 2023-11-21

**Authors:** Kim L. Bennell, Rachel K. Nelligan, Michelle Hall, Sarah Stratulate, Fiona McManus, Karen Lamb, Jennifer Marlow, Rana S. Hinman

**Affiliations:** 1 Centre for Health, Exercise and Sports Medicine, Department of Physiotherapy, The University of Melbourne, Melbourne, Vic Australia; 2 Centre for Epidemiology and Biostatistics, Melbourne School of Population and Global Health, The University of Melbourne, Melbourne, Vic Australia

**Keywords:** Osteoarthritis, Hip, Rehabilitation, Telehealth, Internet, Digital, Exercise, Physical activity, Clinical trial, RCT, Pain

## Abstract

**Background:**

Hip osteoarthritis (OA) is a leading global cause of chronic pain and disability. Given there is no cure for OA, patient self management is vital with education and exercise being core recommended treatments. However, there is under-utilisation of these treatments due to a range of clinician and patient factors. Innovative service models that increase patient accessibility to such treatments and provide support to engage are needed. This study primarily aims to determine the effects of a self-directed digital exercise intervention comprising online education and exercise supported by a mobile app to facilitate adherence on the primary outcomes of changes in hip pain during walking and patient-reported physical function at 24-weeks when compared to online education control for people with hip OA.

**Methods:**

We will conduct a two-arm, superiority parallel-design, randomised controlled trial involving 182 community volunteers aged 45 years and over, with painful hip OA. After completing the baseline assessment, participants will be randomly assigned to either: i) digital exercise intervention; or ii) digital education (control). Participants randomised to the intervention group will have access to a website that provides information about hip OA and its management, advice about increasing their physical activity levels, a 24-week lower limb strength exercise program to be undertaken at home three times per week, and a mobile app to reinforce home exercise program adherence. Participants in the control group will have access to a website containing only information about hip OA and its management. All participants will be reassessed at 24 weeks after randomisation. Primary outcomes are severity of hip pain while walking using an 11-point numeric rating scale and physical function using the Western Ontario and McMaster Universities Osteoarthritis Index subscale. Secondary outcomes are the Hip dysfunction and Osteoarthritis Outcome Score subscales of pain, hip-related quality of life, and function, sports and recreational activities; global change in hip condition; health-related quality of life; measures of physical activity levels; fear of movement; self efficacy for pain and for exercise; and use of oral pain medications.

**Discussion:**

Innovative and scalable approaches to OA education, physical activity, and exercise are required in order to improve exercise participation/engagement and mitigate physical inactivity in the hip OA population. This will help minimise the burden of this major public health issue on individuals and society.

**Trial registration:**

Australia New Zealand Clinical Trials Registry** (**ACTRN12622001533785).

## Background

Osteoarthritis (OA) is a leading cause of chronic pain and disability globally [1] with hip OA affecting one in four adults in their lifetime [2]. As there is no cure, supporting people to self-manage is key in the treatment of OA. Clinical guidelines recommend education and physical activity, including structured exercise, as the foundation of conservative management, regardless of disease stage [3]. Evidence shows that exercise improves physical function, hip pain and quality of life [4] and reduces the need for hip replacement in people with hip OA [5]. However, evidence-practice gaps exist in OA [6], with under-utilisation of non-drug treatments, such as exercise, a global problem [7].

Undertaking regular exercise requires long-term behaviour change [8] and is often difficult for people with OA. Many are not given the advice or support needed to integrate appropriate exercises into their daily routine [6, 9]. A number of factors may contribute. General practitioners often report a lack of knowledge, skill and/or time to prescribe exercise [10, 11]. Clinicians, such as physiotherapists and exercise physiologists, who have the necessary knowledge and skills to prescribe exercise programs are often not referred to [6], can be challenging for patients to access due to cost and geographical location [12], and if consulted may have insufficient training or skills in behaviour change techniques to encourage longer-term exercise adherence [13, 14]. Finally, individuals with OA may not have the necessary information and confidence to start, progress and adhere to exercise [12], and often hold unhelpful beliefs about OA that affect their acceptance of nonsurgical, evidence-based treatments such as exercise [15, 16].

Digital interventions may be one scalable method to overcoming some of the barriers associated with exercise prescription and uptake in people with OA. Several OA-targeted digital exercise interventions have been evaluated, mostly in people with knee OA or in mixed knee and hip OA samples, with none specifically in those with hip OA [17–24]. These interventions have been generally well received by people with OA and show promise for clinical outcomes. However, some of these programs require health professional or administrative involvement and are not fully self-directed [18, 20, 22, 24]; require substantial input by users for program individualisation [18, 22, 24], which can be burdensome; do not include resistance/strength training protocols [17]; are not freely available [22–24]; have not been specifically informed by behaviour change theory; and/or lack support to facilitate engagement and long-term exercise behaviour change [17].

We previously developed a 24-week self-directed digital intervention specifically for people with knee OA consisting of website-delivered OA education, physical activity guidance and home-based strengthening exercise (www.mykneeexercise.org.au), supported by a fully automated behaviour change text message exercise adherence program [25] based on the Behaviour Change Wheel model [26, 27]. Our randomised controlled trial (RCT) of 206 patients with knee OA [28] found larger improvements in knee pain and physical function in the intervention group compared with the control at 24-weeks, with most secondary outcomes also favouring the intervention group. Furthermore, a qualitative study revealed that the digital program was easy to use, convenient, effective, helped with confidence to self-manage and provided a sense of support and accountability [29]. We have now made the ‘My Knee Exercise’ program (mykneeexercise.org.au) freely available. To increase scalability of the behaviour change text message program, we also re-developed the texts into a mobile device application (app), available via the Apple App Store and Google Play [30]. The mobile app also incorporates other behaviour change strategies that were not suited to text messaging, such as a graphics to enable self monitoring of exercise behaviour/adherence.

Based on the success of our self-directed digital intervention for those with knee OA, we will now evaluate a similar intervention for people with hip OA. The intervention is modified from the ‘My Knee Exercise’ program and includes educational information, physical activity guidance and exercises appropriate for hip OA as well as the app to monitor and support exercise participation. It requires evaluation in people with hip OA as results from knee OA cannot necessarily be generalised given differences in response to exercise and patient characteristics [31]. The primary aim of this study is to determine the effectiveness of the digital intervention on primary outcomes of change in hip pain while walking and change in physical function after 24 weeks, compared with a digital education-only control, for people with hip OA. We also aim to determine whether the program will improve secondary outcomes at 24 weeks.

## Methods

### Study design

This is a two-group, parallel-design, superiority RCT conducted across Australia. The trial is designed according to SPIRIT (Standard Protocol Items: Recommendations for Interventional Trials) guidelines [32] and principles of Good Clinical Practice. It has been prospectively registered (ACTRN12622001533785) and will be reported according to the CONSORT statement and relevant extensions [33] as well as TIDieR guidelines [34]. There are no planned interim analyses or stopping guidelines. We will record any protocol amendments in our trial protocol document, notify the institutional ethics committee and update the trial registry if appropriate.

### Participants

We will recruit community participants from across Australia. A total of 182 participants with chronic hip pain consistent with a clinical diagnosis of hip OA will be recruited via media, social media, email newsletters, and from our consumer network.

Inclusion criteria are as follows:

i) National Institute for Health and Care Excellence [35] clinical criteria for OA:
  age ≥ 45 years;
  activity-related hip joint pain; and
  morning hip stiffness ≤ 30 min or no morning hip stiffness;
ii) Hip pain for ≥ 3 months;
iii) Hip pain on most days in the past month;
iv) Average hip pain during walking in past week as ≥ 4 out of 10 on an 11-point numeric rating scale (NRS);
v) Home internet connection, a computer/tablet device with internet access and a suitable phone to download an app; and
vi) Able to participate fully in the intervention and assessment procedures and provide informed consent.

Exclusion criteria are as follows:

i) Hip joint replacement in the more painful hip;
ii) Planning to undergo a hip joint replacement in the next 6 months;
iii) Participation in regular leg strengthening exercise over the past 6 weeks (one or more times per week for each week);
iv) Self-reported diagnosis of an inflammatory arthritic condition, such as rheumatoid arthritis;
v) Fall within the last 12 months and do not receive clearance from a general practitioner to participate in an unsupervised home exercise program;
vi) Housebound due to mobility limitations and unable to leave the house in the last month without assistance from another person and do not receive medical clearance from a general practitioner to participate in an unsupervised home exercise program;
vii) Health condition(s) listed on the Exercise and Sports Science Australia stage 1 pre-exercise screening questionnaire that might compromise exercise safety [36] and do not receive medical clearance from a general practitioner to participate in an unsupervised home exercise program; and/or
viii) Unable to read or speak English.

### Procedures overview

**Fig. 1:**
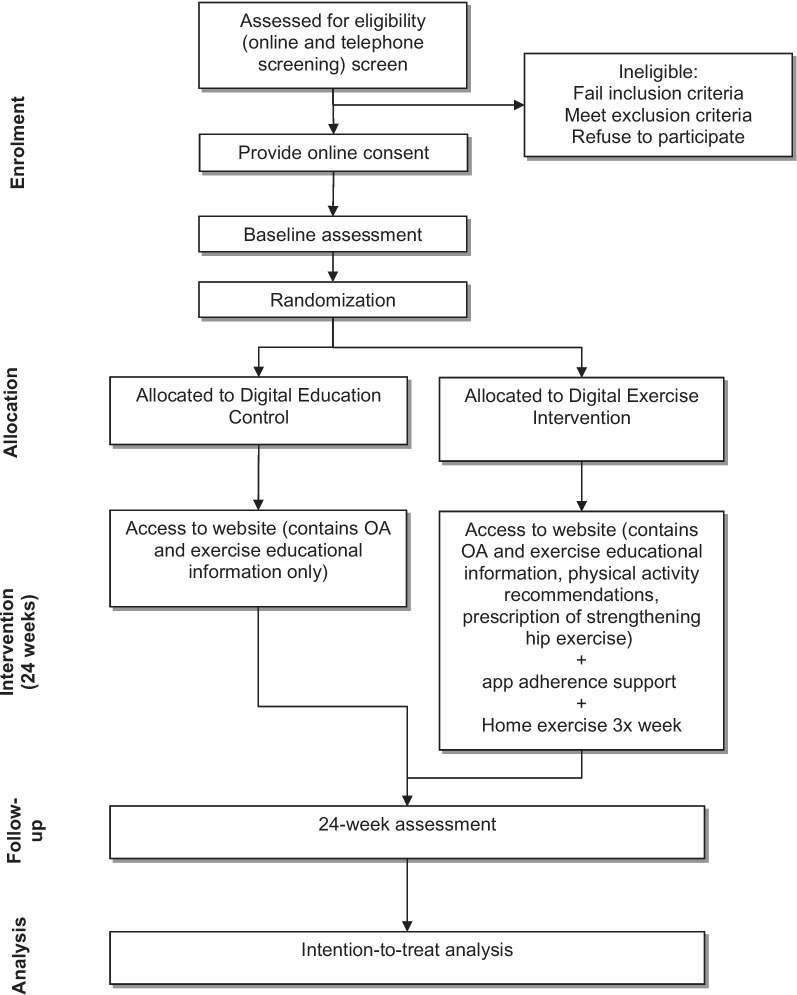
Participant flow through the randomised controlled trial

Figure 1 summarises the trial phases. Volunteers will complete an online questionnaire, with those passing initial screening contacted by a research team member by telephone to undergo further screening and to be provided with verbal information about the study. Eligible volunteers will then be emailed the Plain Language Statement and will be asked to contact the researchers if they have any questions or concerns about its contents. Prior to completion of baseline questionnaires, informed consent will be obtained from all participants online using REDCap™.

For participants with bilateral hip pain, the more painful eligible hip will be deemed the study hip with respect to the exercise intervention and outcome measurement. If both hips are equally painful and eligible, the right hip will be deemed the study hip. Participants will not be advised against accessing co-interventions during the study timeframe, and usage of co-interventions will be recorded as described below.

### Randomisation, blinding and allocation concealment

Eligible participants will be randomised in a 1:1 ratio to either the i) digital exercise intervention, or ii) digital education (control). The randomisation schedule will be computer generated by an independent biostatistician, using permuted random block sizes. The randomisation schedule will be stored at the University of Melbourne on a password-protected website (REDCap™) and maintained by another researcher who is not involved in the recruitment of participants or administration of primary/secondary outcome measures. After the baseline assessment has been completed, group allocation will be revealed by this same researcher.

Limited disclosure will be used to blind participants to group allocation. At the time of recruitment, participants will be informed that the study will investigate and compare digital resources/websites designed to help people self-manage their hip pain and that this could include exercise and online resources. Given participants are blinded and outcomes are self-reported measures, this constitutes assessor blinding. Research staff administering and entering any participant-reported data will be blinded. The statistical analysis plan will be written while the biostatisticians are blinded. Main statistical analyses will be performed blinded to group name.

### Interventions

#### a) Digital exercise intervention

Participants will receive access to a bespoke website and a 24-week exercise adherence support mobile app. Immediately post-randomisation, the research team will send an email to participants with details about how they can log into the website (the website URL and their unique username and password) and download the app. Participants will be asked to log into the website on their own device within 7 days and informed they can then access the website as little or as often as they wish. A welcome text message will also be sent prompting participants to check their email to access the website.

### My hip exercise website

The website is based on our “My Knee Exercise” website (www.mykneeexercise.org.au). The website is comprised of a home page as well as five different tabs containing educational information (My Hip Education), a 24-week lower limb strength home exercise program and supplemental resources (ie. logbooks) to support the program (My Hip Strength), guidance for increasing general physical activity (My Hip Activity), a synthesis of resources from within the website (My Hip Tools), and information about an exercise adherence support app (My Exercise Messages). The website was created by the authors and constructed in line recommendations from The Health on the Net Foundation’s Code of Conduct [37]. The readability levels of all written material in the website were evaluated with recommended [38] and previously used [39, 40] online literacy software (Readable.io, Added Bytes, Ltd, Sussex, UK) as appropriate for people with a 7-8th-grade reading level. This is within the suggested reading ability for consumer healthcare information [41, 42]. Website sections are described in depth below.

#### Home page

The website main page includes an instructional video which explains use of each page of the website, recommends participants start the strength exercises as soon as possible and describes the app and other resources for exercise support. The home page also includes links to other sections of the website and to ‘contact us’ and ‘about us’ information.

#### My hip education

This section encompasses written advice about living with hip OA, hip OA treatments, exercise as a hip OA treatment, and coping with exercise pain. Videos are used to support written information, with interviews of people with hip OA and of OA experts. Participants are encouraged to review the text and multi-media material prior to beginning the ‘My Hip Strength’ prescribed strengthening exercise program. It also recommends two other free online programs that provide OA education (www.futurelearn.com/courses/taking-control-hip-and-knee-osteoarthritis) and pain coping skills training (www.paintrainer.org) that participants may choose to access if they wish.

#### My hip strength

This section houses the 24-week, home-based, self-directed, strengthening exercise program (comprising three sequential progressive programs each lasting 8 weeks). Exercises target the lower limb muscles. Dosage for most exercises is: 10 repetitions; three sets; three times a week; at a “hard” exercise intensity (5–6) to “very hard” (7–8) as rated on an 11-point scale of Rated Perceived Exertion (RPE) for strength training [43]. The difficulty of each exercise can be increased by adding ankle weights and/or changing body position. Participants are encouraged to source their own weights to increase their exercise difficulty over the course of the 24-week program, in line with a pragmatic approach. Exercise sessions are recommended to last about 20–30 min each. Each exercise is accompanied by written instructions as well as instructional images and videos. Each exercise program is able to be downloaded and printed. Printable exercise logbooks for each program (1, 2 and 3) are provided for people to record their weekly exercise practice if they desire. This section also contains advice about purchasing exercise equipment to progress the exercises, tips for starting and adhering to exercise, and suggestions for managing exercise pain.

#### My hip physical activity

This page contains information and guidance to help participants increase their physical activity over the 24-week period. Information such as why and how to increase physical activity levels, how to monitor and safely progress daily step count, pacing physical activity for hip pain and how to plan general physical activity. A physical activity plan template is available for download as a PDF file. Video interviews are also included within this section where people with hip OA discuss their experiences of increasing physical activity levels in the setting of hip pain.

#### My hip tools

This section contains links to key website sections, including the physical activity plan template, strength exercise program, and physical activity logbooks. The exercise equipment recommendations, tips for commencing and adhering to exercise and the hip OA video interviews are also included.

#### My exercise messages

This section contains information about an exercise adherence support app “My Exercises Messages” which is available for free in the App Store (Apple devices) and Google Play Store (Android devices) [30]. Participants will be instructed to download and use the app within 7 days post-randomisation and to use it for the duration of the 24-week program.

The app was modified from our fully automated behaviour change text message exercise adherence program which we specifically designed for people with hip and/or knee OA [25]. The app works by tracking weekly exercise sessions and providing tailored messages to help overcome obstacles to exercise. Participants will be instructed to set a strengthening exercise target of three times per week in the app (aligned with the recommendations in the My Hip Exercise section). Each week (or fortnight as they progress through the program) they will receive notifications prompting them to use the app to record how many days they performed the exercises in the past week (0–7) and any obstacles encountered. Participants will then receive messages tailored to help them adhere to the strengthening exercise program. Participants will also receive additional messages throughout the week to remind and support them to achieve their strengthening exercise target (3 days/week).

### b) Digital education (control)

Participants in the control group will be provided with a website URL to access a bespoke website that contains the same educational information from the “My Hip Education” section of the intervention website, minus the information and links to the two other free online programs (“Taking Control of Your Hip and Knee Osteoarthritis” and ‘PainTrainer”). It will also contain general exercise and physical activity guidance like that available in current online Australian OA consumer resources, but the site does not contain a strengthening exercise program. All website materials have been developed specifically for this study. Immediately post-randomisation, participants will receive an email from the study co-ordinator with information of how to log into the website (the website URL and their unique username and password). Participants will be instructed to access the website on their personal computer or tablet within 7 days, review any educational information provided and implement any recommendations as they see fit. Participants will be advised that they can access the website as little or as often as they wish. At the same time they receive their access email, participants will also receive a welcome text message prompting them to access the website.

### Outcome measures

Table 1 lists all descriptive data, primary and secondary outcomes, and other measures. Participant-reported outcomes will be collected online via REDCap data capture platform and, unless otherwise indicated, collection is at baseline and 24 weeks after randomisation. Some process measures are also collected at 8 and 16 weeks.

**Table 1:** Schedule of enrolment, intervention and assessments

	**Enrolment**	**Allocation**	**Secondary**		**Primary**
**Timepoint**		***0w***	***8w, 16w***		***24w***
**Enrolment:**					
Eligibility screen	X				
Informed consent	X				
Allocation		X			
**Interventions:**					
Control education website (control group only)					
My Hip Exercise website (intervention group only)					
My Exercise Messages App (intervention group only)					
**Assessments:**					
**Descriptive data**					
Age (years)	X				
Sex	X				
Gender	X				
Height (self-reported, metres)	X				
Weight (self-reported, kilograms)	X				
BMI (calculated from self-reported height and weight)	X				
Ethnicity	X				
Geographical location (postcode)	X				
Education level	X				
Current employment status	X				
Comorbidities (Self-Administered Comorbidity Questionnaire) [40]	X				
Symptoms in other joints	X				
Duration of symptoms (years)	X				
Medication usage	X				
**Primary outcomes**					
Severity of hip pain during walking (11-point NRS)	X				X
Physical function subscale of the WOMAC [44]	X				X
**Secondary outcomes**					
Pain subscale of HOOS [45]	X				X
Function, sports and recreational activities subscale of HOOS [45]	X				X
Hip-related quality-of-life subscale of HOOS [45]	X				X
Global rating of overall change in hip condition					X
Health-related quality of life (AQoL-8D) [46]	X				X
Incidental and Planned Exercise Questionnaire (IPEQ-W) [47]	X				X
Global rating of change in physical activity					X
Brief Fear of Movement Scale for OA [48]	X				X
Pain subscale of the Arthritis Self Efficacy Scale [49]	X				X
Self-efficacy for exercise scale [50]	X				X
Use of oral pain medications for hip pain					X
**Process measures**					
Website visits ^a^			X		X
Downloaded exercise programs (intervention group only) ^b^					X
Number of days home strengthening exercise performed (intervention group only) ^c^			X		X
Exercise Adherence Rating Scale [EARS] Section B (intervention group only)					X
Downloaded “My Exercise Messages” app (intervention group only)					X
Frequency of entering exercise into the “My Exercise Messages” app (intervention group only) ^d^					X
Use of the “My Exercise Messages” app (intervention group only) ^e^			X		X
Usefulness of the “My Hip Exercise” website (intervention group only) ^f^					X
Usefulness of the “My Exercise Messages” app (intervention group only) ^g^					X
Exercise equipment use (intervention group only) ^h^					X
Registration to recommended external websites (intervention group only) ^i^					X
**Other measures**					
Co-intervention use					X
Leg strengthening exercises in the past 24 weeks (control group only)					X
Adverse events					X

*WOMAC* Western Ontario and McMaster Universities Osteoarthritis Index, *BMI* Body Mass Index, *OA* Osteoarthritis, *NRS* Numeric rating scale, *HOOS* Hip dysfunction and Osteoarthritis Outcome Score

^a^Self-reported. Participants will be asked “How many times did you visit the website over the past 2 months?” with response options: “never”, “1–5 times”, “6–10 times” and “ > 10 times”

^b^Yes/No response to “Did you download any of the exercise programs from the website as PDF documents?” for programs 1, 2, and 3

^c^Self-reported. Participants will be asked “In the past two weeks, on how many days did you perform the strengthening exercises from the “My Hip Exercise” program (0–14)

^d^Self-reported. Scored on a 5-point Likert scale for “How often did you enter your number of exercise days into the app as requested” (Never, rarely, sometimes, often, always)

^e^Self-reported. Participants will be asked “On how many days in the past 14 days did you use the “My Exercise Messages” app (including reading any notifications, entering data and/or opening the app)” with response options: “never”, “1–5 times”, “6–10 times” and “ > 10 times”

^f^Self-reported. Scored on an 11-point NRS for “How useful did you find the “My Hip Exercise” website program? (not including the app)” from 0 = “not at all useful” to 10 = “extremely useful”

^g^Self-reported. Scored on an 11-point NRS for “How useful did you find the “My Exercise Messages” app?” from 0 = “not at all useful” to 10 = “extremely useful”

^h^Yes/No response to “Did you use exercise equipment (such as weights or elastic bands) to add resistance to your leg for your strengthening exercises?”

^i^Yes/No response to “Did you register for this course?” for 1) an online course “Taking Control of Your Hip and Knee Osteoarthritis,” and 2) an online course “PainTRAINER”

The two primary outcomes are psychometrically acceptable, reliable and valid measures recommended for use in clinical trials of hip OA [51].

i) Average severity of hip pain on walking in the past week measured on an 11-point NRS, where 0 = “no pain” and 10 = “worst pain possible”;

ii) Physical function subscale of the Western Ontario and McMaster Universities Osteoarthritis Index (WOMAC) [44] with scores from 0 to 68, where higher scores indicate greater dysfunction.

Secondary outcomes include:

i) Pain subscale of the Hip dysfunction and Osteoarthritis Outcome Score (HOOS) [45] with 10 items and normalised scores ranging from 0 to 100, with 100 indicating no pain;
ii) Hip-related quality of life subscale of HOOS [45] with 4 items and normalised scores ranging from 0 to 100, with 100 indicating better quality of life;
iii) Function, sports and recreational activities subscale of HOOS [45] with 4 items and normalised scores ranging from 0 to 100, with 100 indicating better function;
iv) Global rating of overall change in hip condition compared to baseline scored on a 7-point Likert scale from “much worse” to “much better” [52] at 24 weeks. Participants who indicate that they are “moderately better” or “much better” will be categorised as ‘improved’;
v) Health-related quality of life using the Assessment of Quality of Life instrument (AQoL-8D) [46], a 35-item instrument with scores ranging from -0.04 to 1.0, higher scores indicating better quality of life;
vi) Incidental and Planned Exercise Questionnaire, version W (IPEQ-W) [47] evaluating physical activity levels in the past week with 10 items and score given as hours per week;
vii) Global rating of change in physical activity compared to baseline scored on a 7-point Likert scale from “much less” to “much more” at 24 weeks. Participants who indicate that they are doing “moderately more” or “much more” will be classified as having increased their amount of physical activity;
viii) Brief Fear of Movement Scale for Osteoarthritis [48] scored from 6 itmes on a 4-point Likert scale with total scores ranging from 6 (minimal fear) to 24 (maximal fear);
ix) Arthritis self-efficacy scale (pain subscale) [49] with comprises 5 items each scored on a 10-point Likert scale and reported as a mean of the items (1–10), higher scores indicating greater self-efficacy;
x) Self-Efficacy for Exercise Scale [50] scored from 9 items on an 11-point NRS from “not confident” to “very confident” with scores ranging from 0–90, higher scores indicating greater self efficacy for exercise;
xi) Use of oral pain medications for hip pain self-reported at 24 weeks and defined as one or more of analgesics (paracetamol combinations) and/or oral non-steroidal anti-inflammatory drugs and/or oral glucocorticoids and/or oral opioids taken at least once a week in the prior month for hip pain.

### Other measures

i) Co-interventions: At 24 weeks, participants will be asked to fill in a custom-devised table indicating their frequency of use of a range of other hip treatments (over the past 24 weeks).
ii) Website visits: Participants will be asked “How many times did you visit the website over the past 2 months?” with response options “never”, “1–5 times”, “6–10 times” and “ > 10 times” at 8, 16 and 24 weeks;
iii) Leg strengthening exercise (control group only): At 24 weeks, participants will be asked “Over the past 24 weeks, did you perform leg strengthening exercise one or more times a week for at least 8 weeks?” with response options yes/no.

The intervention group will be asked additional questions at 24 weeks, unless otherwise stated, including:

i) Downloaded the three exercise programs: response options of yes/no for each;
ii) Number of days strengthening exercises performed: Participants will be asked “In the past two weeks, on how many days did you perform the strengthening exercises from the “My Hip Exercise” program (0–14)” at 8, 16 and 24 weeks. Responses will be summed over the three time points and reported as the average number of days per week;
iii) Exercise Adherence Rating Scale (EARS) Section B [53] scored from 6 items with scores ranging from 0–24, higher scores indicating better adherence;
iv) Downloaded My Exercise Messages app: response options of yes/no;
v) Frequency of entering exercise information into app: Scored on a 5-point Likert scale for “How often did you enter your number of exercise days into the app as requested” with response options of never/ rarely/sometimes/often/always;
vi) Use of the app: At 8, 16 and 24 weeks, participants will be asked “On how many days in the past 14 days did you use the “My Exercise Messages” app (including reading any notifications, entering data and/or opening the app)” with response options “never”, “1–5 times”, “6–10 times” and “ > 10 times” at 8, 16 and 24 weeks;
vii) Usefulness of the website: Scored on an 11-point NRS from 0 = “not at all useful” to 10 = “extremely useful”;
viii) Usefulness of the app: Scored on an 11-point NRS from 0 = “not at all useful” to 10 = “extremely useful”;
ix) Exercise equipment use: Participants will be asked “Did you use exercise equipment (such as weights or elastic bands) to add resistance to your leg for your strengthening exercises?” with response options of yes/no;
x) Registered for two recommended external website programs (“Taking Control of Your Hip and Knee Osteoarthritis” and ‘PainTrainer”): response options of yes/no for each.

### Descriptive measures

Baseline self-reported descriptive measures include age, sex, gender, height, weight, body mass index (calculated from height and weight), ethnicity, geographical location determined based on residential postcode, education level, employment status, comorbidities assessed using the Self-Administered Comorbidity Questionnaire [40], symptoms in other joints, hip symptom duration, and pain medication usage.

### Adverse events

Related adverse events will be defined as “any problem experienced in the study hip or elsewhere in the body deemed to be a result of participating in the trial and at least one of i) caused negative/adverse symptoms/effects for two days or more, and/or ii) resulted in the participant seeking treatment or taking medication”. Adverse events will be ascertained by survey questions to participants at 24 weeks.

A serious adverse event is defined as any untoward medical occurrence that; i) results in death; ii) is life-threatening; iii) requires hospitalisation or prolongation of existing inpatient hospitalisation; iv) results in persistent or significant disability or incapacity; v) is a congenital anomaly or birth defect, or; vi) any other important medical condition which, although not included in the above, may require medical or surgical intervention to prevent one of the outcomes listed. Due to the low-risk nature of the interventions in this trial, related serious adverse events are extremely unlikely. Participants will be advised to report any serious adverse events to the Trial Coordinator as soon as they can by telephone or email, which will be documented and reported to the Sponsor (University of Melbourne) within 24 h of the research staff becoming aware of the event. Any adverse events will be reported to the internal Trial Monitoring Committee who will be responsible for deciding on a case-by-case basis what action, if any, is required and whether the adverse events are likely to be related to the intervention.

We will report the number and proportion of participants who: withdraw from the study due to a related adverse event; experience one or more serious related adverse events and their types; and experience one or more non-serious related adverse events and their types.

### Sample size 

We based the sample size on detecting a difference in change between-groups that meets or exceeds a specified minimal clinically important difference (MCID) for the two primary outcomes of hip pain on walking and WOMAC physical function. The MCIDs we used were 1.8 units for NRS hip pain [54] and 6 units for WOMAC physical function [55]. To achieve 80% power and a two-sided 5% significance level split equally across the two primary outcomes, assuming equal between-participant standard deviations of 2.5 for pain and 13 for function for both groups and a correlation between pre- and post-measurements of 0.25 for pain and 0.40 for function [56], and accounting for 15% loss to follow up, we required 91 participants per arm, for a total of 182 participants. We will consider the intervention to be effective if at least one of the two primary outcomes shows a significant between-group difference.

### Data analysis plan

An a priori statistical analysis plan will be published on the Centre for Health, Exercise and Sports Medicine’s website. A statistician blinded to group details will perform analyses comparing the two groups. An intention-to-treat analysis will be conducted using data available from all randomised study participants. Multiple imputation will be conducted, and the method reported if the amount of missing data for either primary outcome is greater than 5%. The primary analysis will then use multiply imputed datasets, with a sensitivity analysis using complete case datasets.

A summary of participant demographics and baseline characteristics will be presented. Linear regression models, adjusted for baseline levels of the outcome, will be used to compare mean differences in change (baseline minus follow up) for continuous variables. Standard diagnostic plots will be used to assess model assumptions. Each primary outcome will also be dichotomised into those who do and do not achieve the MCID in improvement in hip pain on walking and function to aid clinical interpretation of results. For these and other binary outcomes, groups will be compared using risk differences and risk ratios, calculated from logistic regression models and adjusted for the outcomes at baseline where available.

### Patient and public involvement

End-users and stakeholders were engaged in developing the research question, study methodology, and intervention components. A consumer with hip OA (JM) provided input into the research question and study protocol, website usability, the design of the hip strengthening exercise program and the length and ease of access of the initial proposed questionnaire battery. Another consumer and five physiotherapists also provided specific input into the design of the hip strengthening exercises, while one consumer participated in the filming/production of the exercise videos and the images used in the downloadable exercise booklet. Four consumers with hip OA completed usability testing of the website prototype using a think aloud approach which informed the final website. The “My Exercise Messages” app had extensive iterative engagement during the development of the behaviour change message library and app which has been previously described [30].

### Timelines

Ethical approval for the trial was given by The Human Research Ethics Committee of The University of Melbourne on 19^th^ September, 2022. We prospectively registered the trial with the Australian New Zealand Clinical Trials Registry on 12^th^ December, 2022. Participant recruitment started in March 2023. Recruitment is anticipated to be completed in September 2024. The main trial is scheduled for completion in March 2025 with all participants completing 24 week data collection.

### Dissemination

We will disseminate study findings through conference presentations and publication in peer-reviewed journals as well as via our Centre website, knowledge translation network, media and social media, including a study infographic. We will follow the International Committee of Medical Journal Editors recommendations for authorship.

## Discussion

This protocol describes the background, aims and methods for a two-group, parallel design RCT aiming to evaluate the effectiveness of a self-directed unsupervised digital exercise intervention on the two primary outcomes of change in hip pain while walking and physical function at 24 weeks, compared with a digital education-only control for people with hip OA. The effects of the intervention on other clinical outcomes at 24 weeks will also be evaluated. A range of measures will provide information about the safety and acceptability of, and engagement with, this program that focuses on core recommended hip OA treatments of education and exercise/physical activity. Such a program has the potential to enhance patient access to evidence-informed lifestyle treatments and reduce the personal and societal burden of OA.

## Data Availability

The datasets used and/or analysed during the current study will be made available from the corresponding author on reasonable request.
